# Gender influence on the MVV / FEVı ratio in a population of healthy young adults

**DOI:** 10.14814/phy2.14623

**Published:** 2020-10-28

**Authors:** Soualiho Ouattara, Edwige Siransy‐Balayssac, Aya Liliane Kondo, Téniloh Augustin Yéo, Cyrille Serges Dah, Pascal Bogui

**Affiliations:** ^1^ Laboratoire de Physiologie et d’Explorations Fonctionnelles Unité de Formation et de Recherche en Sciences Médicales Université Félix Houphouët Boigny Abidjan Côte d’ivoire; ^2^ Service des Explorations Fonctionnelles Centre hospitalier universitaire de Yopougon Abidjan Côte d’ivoire; ^3^ Service des Explorations Fonctionnelles Centre hospitalier universitaire de Cocody Abidjan Côte d’ivoire

**Keywords:** adult, gender, VMM/FEVı

## Abstract

**Introduction:**

Maximal voluntary ventilation (MVV) and flow expiratory volume in the first second (FEVı) are important spirometric parameters. They are both gender‐dependent. However, estimating the MVV, which is widely practiced in cardiopulmonary function testing, by multiplying FEVı by a constant value (equal to MVV/FEVı ratio) does not seem to take this into account.

The purpose of this study was to compare the MVV/FEVı ratio by gender among healthy young adults.

**Methods:**

This cross‐sectional prospective study involved 67 medical sciences students, including 36 females of the same race, height, and age group. Their ventilatory function was assessed using a computerized spirometer, according to international recommendations.

Pearson's test made it possible to correlate different spirometric parameters and linear regression was established between MVV and FEVı.

The nonparametric Kruskal–Wallis test was used to compare the MVV/FEVı ratio between females and males.

Comparisons by gender were made also between our data and previous prediction equations.

**Results:**

In both females and males, FEVı was the spirometric parameter with which MVV had the highest correlation (*r* = .91 in females, *r* = .63 in males).

A comparison of the means of the MVV/FEVı ratio by gender showed a statistically significant (*p* < .005) decrease in females (35.68 vs. 38.87).

The previous prediction equations showed statistically significant under and overestimation of MVV values when gender was not taken into account.

**Conclusion:**

For the same height, age, and race, the ratio MVV/FEVı was significantly lower for females. So, the use of a preset constant value in estimating the MVV without taking gender into account was methodologically questionable.

This work, which could have clinical implications, would benefit from being confirmed in a larger population.

## Graphical abstract

MVV/FEV1 ratio was 35.68 in females and 38.87 in males, significantly different (*p* = .005). Thus, estimating MVV value should take gender into account.

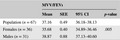

## 1 INTRODUCTION

Spirometry is invaluable as a screening test of general respiratory health (Miller et al., 2005). Indeed, it is used to measure volumes and flow rates of ventilated air, including the maximal voluntary ventilation (MVV), which is the maximum amount of air that can be inhaled or exhaled within one minute, under BTPS conditions. Expressed as liters per minute, MVV reflects respiratory muscle strength and chest wall compliance (Assaf et al., 2017).

There is an interest in measuring MVV for several reasons. As it affects the performance with which a subject inhales or exhales room air, MVV allows for measuring ventilatory reserve during cardiopulmonary exercise testing (CPET), which is necessary to assess ventilatory limitation (Wasserman et al., 1987). It is also useful in the diagnosis of obstructive respiratory diseases (COPD, asthma, cystic fibrosis), in the monitoring of respiratory diseases and in the evaluation of response to treatment (Benditt et al., 1997; Dugan et al., 1995).

In practice, the MVV is measured over 12 s and then extrapolated to one minute to overcome the difficulties (shortness of breath, fatigue, dizziness) that a subject may encounter during the measurement process, which can often be tedious and time‐consuming (Colwell & Bhatia, 2017; Kennedy, 1953). Better still, these difficulties led several authors to develop and use an equation to estimate its value based on maximum volume of air exhaled during the first second of forced expiration (FEVı) following maximum inspiration: MVV = FEVı × 35 (Gandevia & Hugh‐Jones, 1957) FEVı × 37.5 (Cara, 1953) or MVV = FEVı × 40 (Kennedy, 1953) are among the most common in the literature.

Indeed, MVV correlates well with FEVı, which is one of the main spirometric parameters in the diagnosis of bronchial obstruction (Pellegrino et al., 2005) and easier to measure in clinical practice (Miller et al., 2005).

For more than half a century, several authors have been using the constant MVV/FEVı ratio to estimate MVV in any population (Anderson et al., 2001; Colwell & Bhatia, 2017; Wasserman et al., 1987). For example, the Medical Commission of the International Olympic Committee, in its fight against doping in sport, recommends bronchial provocation by eucapnic voluntary hyperventilation (EVH), considered to be the gold standard for diagnosing exercise‐induced bronchoconstriction (EIB). The EVH protocol required the athlete to hyperventilate for 6 min at a target minute ventilation (TV) equal to 85% of a VMM which has been estimated by FEVı times 35 (Anderson et al., 2001). This protocol has been in use for about 20 years now in athletes (Koch et al., 2015; Spiering et al., 2004) and even in patients (Campbell, 1982).

This approach appears problematic because it obscures the fact that the constant value of the MVV/FEVı ratio used in all these studies appears to have been determined without taking gender into account. However, many authors have shown that these two ventilatory flow rates were gender‐dependent in both leukoderm and melanoderm populations (Musafiri et al., 2013; Pellegrino et al., 2005).

The following question, therefore, deserved to be asked: is the estimation of the MVV using a previously fixed MVV/FEVı ratio correct regardless of subject gender? Hence, the aims of our study were to evaluate the influence of gender on the value of the FEVı/MVV ratio in healthy young adults.

## 2 MATERIALS AND METHODS

### 2.1 Ethical approval

This study was conducted in accordance with the guidelines set by the Declaration of Helsinki and was approved by the Ethics Committee of Abidjan Medical Sciences Research unit (Ivory Coast).

All subjects were volunteers and advised about the purpose and procedures of the study, and gave their informed consent.

### 2.2 Population

This study was carried out from May 2014 to December 2015 in the physiology and functional explorations laboratory of the Training and Research Unit of Medical Sciences in Abidjan, Ivory Coast. Among the medical science students, 67 subjects including 36 females were selected based on the following inclusion criteria: 20 to 25 years of age, black African origin, height between 1.60 and 1.70 m, and a body mass index (BMI) between 18.5 and 24.9 kg/m^2^ sedentary, nonsmokers and showed no cardiovascular, respiratory, or hematological disease.

None of the students was affected by the exclusion criterion, which was the poor execution of spirometric maneuvers.

### 2.3 Protocol study

The spirometry test was performed in accordance with ATS/ERS guidelines (Miller et al., 2005) by a trained respiratory technician. The device used was a computerized spirometer “microQuark” (Cosmed; Italy). During the appointment time between 3 p.m. and 6 p.m., subjects were comfortably seated on a chair, resting for 15 min in a well‐ventilated room, at a temperature of 24°C to 27°C (degree Celsius) and a relative hygrometry of 75% to 90%. They were advised not to drink coffee or tea and not to engage in any physical activity on the day of testing. They were also advised to wear loose clothing to allow full chest and abdominal expansion.

Before initiating the test, an explanatory phase led by the technician allowed the subject to become familiar with the objectives and the course of the test and to become familiar with the spirometer through two or more tests. This phase was essential to ensure the quality of the steps.

The spirometric maneuvers were carried out in three stages under visual control of curves displayed on the spirometer screen:

First, obtaining the volume‐time curve for the measurement of vital capacity (VC) components (tidal volume, inspiratory reserve volume, and expiratory reserve volume). The subject, with nose clip‐on, successively performed calm breathing, then a slow, gentle, maximal inspiration followed by a slow, gentle, maximal expiration. Three tests were carried out within 1‐min interval and the best curve was selected.

Next, the flow‐volume curve was obtained for the measurement of forced vital capacity (FVC). This procedure consisted of three phases: calm breathing then a fast maximal inspiration followed by a “blast,” continued complete exhalation for at least 6 s. The parameters FVC, FEVı, peak expiratory flow (PEF), instantaneous forced expiratory flow at different percentages of FVC (FEF75, FEF50, FEF25) and mean forced expiratory flow between 25% and 75% of FVC (FEF25%‐75%) were automatically evaluated and expressed in relation to their predicted value.

For this FVC measurement, the curve with the highest FEVı + FVC sum after three satisfactory maneuvers, was the best. All parameters measured on this curve were retained.

Finally, for the MVV measurement, the subject was instructed to breathe normally at first and then, when the technician gave the signal, he had to inhale and exhale as fast and as fully as possible. The test was performed over 12 s and then extrapolated to one minute. The measured value was given directly in liters per minute with the percentage of the predicted value.

Three maneuvers (or more if the MVV values varied by more than 15 liters/min) were carried out with an interval of 3 min and the best was selected taking into account the MVV/FEVı X 40 > 0.8 criterion (Campbell, 1982; Miller et al., 2005).

## 3 STATISTICAL ANALYSIS

Anthropometric data (gender, age, height, weight, BMI) and spirometric parameters measured and related to predicted values (FVC, VC, FEVı, and MVV) were presented as averages (± standard deviation).

The statistical analysis was carried out in several stages (data processing, verification, and evaluation), using the EPI INFO software (version 7.2).

The one‐sample Kolmogorov–Smirnov test, for each parameter and in each gender, was used to verify the normal distribution of the data.

This test has shown that all the spirometric parameters measured were normally distributed in the group of males and in that of females, the statistical test used was the Students’ *t*‐test for comparison of means between gender‐based groups.

Pearson's test was used for the determination of correlations between the MVV and other spirometric parameters and anthropometric measurements.

Ratio and linear regression were determined between the MVV and FEVı in each gender.

As for the MVV/ FEVı ratio, it followed the law of normal distribution in the group of males but not in that of females. We, therefore, used the nonparametric Kruskal–Wallis test to compare the ratios between females and males.

The agreement between our prediction equations (from our two ratios) and the two most cited MVV prediction equations from published reports in CPET and EVH testing were assessed: estimated MVV values based on these prediction equations were determined by multiplying the FEVı, acquired during spirometry, by 35 (Gandevia & Hugh‐Jones, 1957), 40 (Kennedy, 1953), and our two MVV/FEVı ratios.

These four estimated MVV values were compared by gender, using the Student's *t*‐test for paired samples.

The mean percentage difference in MVV values between our prediction equations and those of previous studies (Gandevia & Hugh‐Jones, 1957; Kennedy, 1953) is calculated.

The statistical significance was considered as *p* < .05.

## 4 RESULTS

Anthropometric and spirometric parameters are presented in Table 1.

**TABLE 1:** Comparison of anthropometric measurements and spirometric parameters by gender

	Females (*n* = 36)	Males (*n* = 31)
Age (year)	22.31 ± 1.44	22.44 ± 1.19
Weight (kg)	64.41 ± 3.37	65.23 ± 3.04
Height (m)	1.66 ± 0.03	1.67 ± 0.03
B.M.I (kg/m^2^)	23.44 ± 1.16	21.29 ± 2.27
FEVı (l/s) (% pred)	2.60 ± 0.43 (90 ± 15)	3.38 ± 0.40*(99 ± 19)*
FVC (l/min) (% pred)	2.90 ± 0.43 (86 ± 16)	3.87 ± 0.59*(94 ± 13)*
VC (l/min) (% pred)	3.03 ± 0.48 (95 ± 20)	4.01 ± 0.49*(104 ± 21)*
MVV (l/min) (% pred)	93.01 ± 15.74 (92 ± 14)	130.53 ± 21.76*(101 ± 22)*
FEVı/VC (%)	0.86 ± 0.06	0.84 ± 0.05
FEVı/FVC (%)	91 ± 5	89 ± 9

Note

Data expressed as mean ± standard deviation.

Abbreviations: BMI, body mass index kg/m^2^; FEVı, forced expiratory volume in the first second; FVC, forced vital capacity; MVV, maximal voluntary ventilation; VC, vital capacity.

* Significantly different from the value obtained for female (*p* < .05).

The respective mean values of age, height, weight, and BMI of females were statistically similar to those of males.

The means of FEVı, MVV, FVC, and VC were significantly greater for males.

For FEVı/VC and FEVı/FVC, the difference between genders was not significant.

In both females and males, FEVı was the spirometric parameter with which MVV had the highest correlation (Tables 2 and 3).

**TABLE 2:** Correlation between spirometric parameters and anthropometric measurements in females (*n* = 36)

	VMM	Age (years)	Height (m)	Weight (kg)	B.M.I (kg/m^2^)	FVC (L. BTPS)	VC (L.BTPS)
Age (years)	−0.12						
Height (m)	−0.08	−0.16					
Weight (kg)	0.03	−0.06	0.24				
B.M.I (kg/m^2^)	0.09	0.07	−0.57	0.66			
FVC (L. BTPS)	0.88*	−0.10	0.009	−0.07	−0.06		
VC (L.BTPS)	0.88*	−0.14	−0.002	−0.05	−0.04	0.99*	
FEVı (L.s^−1^ BTPS)	0.91*	−0.22	−0.09	−0.10	−0.01	0.92*	0.92*

Abbreviations: BMI, body mass index kg/m^2^; FEVı, forced expiratory volume in the first second; FVC, forced vital capacity; MVV, maximal voluntary ventilation; VC, vital capacity.

* Significantly different from the value obtained for female (*p* < .05).

**TABLE 3:** Correlation between spirometric parameters and anthropometric measurements in males (*n* = 31)

	VMM	Age (years)	Height (m)	Weight (kg)	B.M.I (kg/m^2^)	FVC (L.BTPS)	VC (L.BTPS)
Age (year)	0.11						
Height (m)	0.12	0.03					
Weight (kg)	0.24	0.20	0.37				
B.M.I (kg/m^2^)	0.15	0.16	−0.37	0.72			
FVC (L.BTPS)	0.46*	0.38	0.29	0.46	0.24		
VC (L.BTPS)	0.49*	0.33	0.36	0.43	0.16	0.93*	
FEVı (L.s^−1^BTPS)	0.63*	0.49*	0.32	0.45	0.21	0.80*	0.87*

Abbreviations: BMI, body mass index kg/m^2^; FEVı, forced expiratory volume in the first second; FVC, forced vital capacity; MVV, maximal voluntary ventilation; VC, vital capacity.

* Significantly different from the value obtained for female (*p* < .05).

**FIGURE 1:**
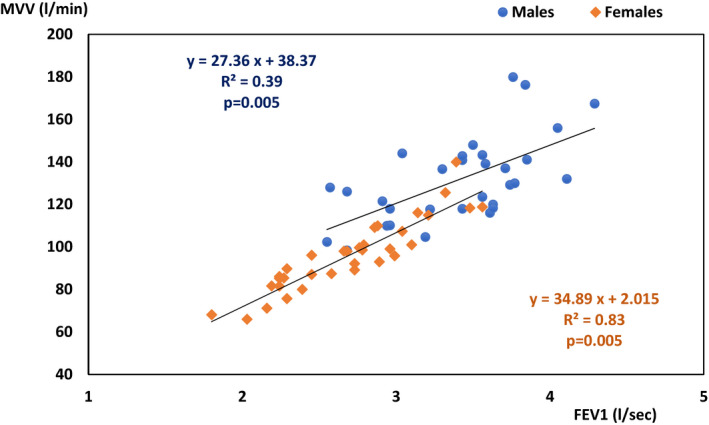
Relationship between the MVV and FEVı measured in females (n = 36) and males (n = 31). FEVı, forced expiratory volume in the first second; MVV, maximal voluntary ventilation

The relationship between measured MVV and FEVı (Figure 1) was statically significant in females (*r* = .91 and *p* < .001) and in males (*r* = .63 and *p* < .001).

The MVV/FEVı ratio was 35.68 in females and 38.87 in males. Thus, the estimated MVV = FEVı × 35.68 or MVV = FEVı × 38.87.

The observed difference between the mean values of these two ratios was statistically significant (*p* = .005). (Table 4).

**TABLE 4:** MVV/FEVı ratios by gender

	MVV/FEVı			
	Mean	SEE	95% CI	*p*‐value
Population (*n* = 67)	37.16	0.49	36.18–38.13	
Females (*n* = 36)	35.68	0.40	34.89–36.46	.005
Males (*n* = 31)	38.87	0.88	37.13–40.60	

Abbreviations: CI, confidence interval à 95%; SEE, standard estimation error.

For the comparisons between prediction equations (Table 5), the estimated MVV values using FEVı times 35 (Gandevia & Hugh‐Jones, 1957) or 35.68 (our ratio in females), were significantly different (*p* = .001) in males but not in females. Conversely, using FEVı times 40 (Kennedy, 1953) or 38.87 (our ratio in males), the estimated MVV values were significantly different (*p* = .001) in females but not in males.

**TABLE 5:** Comparison of estimated MVV values from two commonly cited equations and present study equations

Prediction equation (authors)	Estimated mean MVV (*SD*)	Mean difference % (*SD*)	*t*‐test	*p*‐value
Females				
FEVı X 35 (Gandevia & Hugh‐Jones, 1957)	93.83 (15.17)	−1.93 (6.86)	−1.69	.10
FEVıX 40 (Kennedy, 1953)	107.24* (17.34)	10.80 (6.01)	10.79	<.001
FEVı X 35.68 (present study)	95.66 (15.47)	0.008 (6.73)	0.007	.99
FEVı X 38.87 (present study)	104.21* (16.85)	8.21 (6.18)	7.97	<.001
Males				
FEVı X 35 (Gandevia & Hugh‐Jones, 1957)	119.09* (16.09)	−11.06 (14.11)	−4.37	<.001
FEVı X 40 (Kennedy, 1953)	136.10 (18.39)	2.81 (12.34)	1.27	.21
FEVı X 35.68 (present study)	121.40* (16.40)	−8.94 (13.84)	−3.60	<.001
FEVı X 38.87 (present study)	132.25 (17.87)	−0.007 (12.70)	−0.003	.99

Note

% difference [(percent difference) was calculated as: [(actual MVV‐estimated MVV)/actual MVV] × 100.

* *p* = .001.

The mean percentage difference between our estimated MVV values and the estimated MVV values by FEVı times 40 (Kennedy, 1953) in females was 10.80 ± 6% (*p* < .001). It was −11.06 ± 14.11% (*p* < .001) in males when the MVV values were estimated by FEVı times 35 (Gandevia & Hugh‐Jones, 1957).

## 5 DISCUSSION

The aim of this study was to assess the MVV/FEVı ratio in 67 healthy young adults, including 36 females. The ratio obtained in females was significantly lower than in males (*p* = .005).

This result was in agreement with those of several authors (Giske et al., 2003; Silva et al., 2020). Conversely, others have shown that the ratio was the same, whatever gender (Cid‐Juarez et al., 2017).

Anyway, the major strength of our study was to have reduced the methodological bias that could be present in several of these studies: age of subjects, which often varied by more than two decades (Cid‐Juarez et al., 2017), subjects who were of different sizes (Cid‐Juarez et al., 2017; Giske et al., 2003; Mohan‐Kumar &Gimenez, 1984) or unspecified ethnic origin (Cid‐Juarez et al., 2017; Giske et al., 2003; Mohan‐Kumar & Gimenez, 1984; Silva et al., 2020).

We minimized these possible selection biases related to the influence of anthropometric measurements (age, height, race) on spirometric parameters (Musafiri et al., 2013; Pellegrino et al., 2005).

This methodological constraint had led to one of the limitations of our work, namely the small number of our study population. Indeed, the subject's minimum age was set at 20 years, justified by the fact that it corresponds to the end of the increase in lung function undertaken from birth (Brouard et al., 2011).

The maximum age was set at 25 years, well below the 30‐years of age limit at which lung function declines (Brouard et al., 2011). This limited interval of 20 to 25 years thus minimized the well‐known impact of age on spirometric parameters studied (Gibson et al., 1976).

Similarly, the 67 subjects were not only of the same racial background, but were also selected within a narrow range of height and weight (18.5 kg/m^2^ ≥ BMI≤24.9 kg/m^2^).

All these requirements for subject selection, however, resulted in maximum homogeneity in our sample, which was statistically supported by the absence of significant differences in anthropometric data (age, height, and BMI) between the genders (Table 1), in contrast to several of the studies mentioned above (Cid‐Juarez et al., 2017; Giske et al., 2003; Mohan‐Kumar & Gimenez, 1984; Silva et al., 2020). In each gender, there was also a significant correlation between spirometric parameters (Tables 2 and 3) and a significant relationship between MVV and FEVı (Figure 1).

Thus, our work showed that, for the same height, age and race, the MVV/FEVı ratio was significantly lower for females (Table 4), and the resulting prediction equations were as follows:

MVV = 35.7 × FEVı (females) versus MVV = 38.9 × FEVı (males) with *p* = .005.

An important point to consider was whether the statistically significant difference between the two MVV/FEVı ratios from our study (35.7 and 38.9), was of functional importance.

Comparing the MVV prediction equations from these two ratios (35.7 and 38.9) and those most used in decades by many authors, we showed (Table 5) that the Kennedy's equation (1953) was not applicable to females due to the overestimation of MVV values (*p* = .001). The Gandevia and Hugh‐Jones's equation (1957), neither, could not be applicable to males due to the underestimation of MVV values (*p* = .001).

Similarly, the prediction equations resulting from the ratio determined in females (FEVı times 35.68) were not interchangeable with that obtained from the ratio determined in males (FEVı times 38.87). Indeed, (FEVı times 35.68) underestimated MVV values in males (*p* = .001). and (FEVı times 38.87) overestimated MVV values in females (*p* = .001)..

These results were consistent with those of Neder et al. (1999): after assessing the ventilation limitation in 100 nonsmoking subjects (50 females included), they concluded that the fraction of MVV used for the determination of this limitation should take gender into account.

In practice, a male subject in our study who has a FEVı of 4.29 L/s, his estimated MVV by Gandevia and Hugh–Jones's equation (FEVı times 35) would be reduced by more than 16.5 liter/min compared with the estimated MVV found for our male subjects (FEVı times 38.87). Moreover, its target minute ventilation for bronchial provocation by EVH (EVH TV) would also be reduced by about 14 liters/min (i.e., 10%) for 6 min.

As O’Cain et al. (1980) have shown that the bronchoconstrictive response to EVH is directly proportional to TV, this 10% inaccuracy of the EVH TV for 6 min may lead to a false‐negative diagnosis, especially since the EIB cut off criterion was set of a 10% fall in FEVı (Anderson et al., 2001; Spiering et al., 2004). Therefore, this variability in EVH TV could manifest itself in underdiagnoses of EIB in individual.

This same inaccuracy was found for a female subject in our study with a FEVı of 3.56 L/sec. Her MVV estimated by Kennedy equation (1953) would be increased by 15.38 L/min (3.56 times 40 vs. 3.56 times 35.68), that is, 12%. Such an increase would reduce the diagnostic potential of CPET with an imprecise determination of ventilatory limitation defined as ventilatory reserve < 20% MVV (Colwell & Bhatia, 2017). An overestimation of MVV value would thus lead to an erroneous exclusion of a diagnosis of ventilatory limitation in individuals.

Since the inadequate reference values lead to a practical misinterpretation, a rigorous approach to determining the value of MVV/FEVı ratio by gender was required.

This gender difference of MVV/FEVı ratios could be explained by arguments from the literature:

Firstly, genetic determinants of both FEVı and MVV were different. In fact, as for several spirometric parameters, heritability was involved to a greater or lesser extent. When the two rates were expressed according to their predicted value, the share of heritability h^2^ according to some authors was 22% for the MVV and 30% for FEVı (Vasilopoulos et al., 2013). The impact of environmental factors on the phenotypic expression of these two flow rates would also be different (Vasilopoulos et al., 2013), probably contributing to this inequality between the ratios calculated according to gender.

Second, this difference in the MVV/FEVı ratio between female and male with the same racial and anthropometric characteristics suggested probable, although not fully known, links between sex hormones and certain lung functions. Some studies have noted the effect of male hormones in increasing airway conductance and proximal bronchial flow (Townsend et al., 2012).

Third, Gibson et al., (1976) have shown lower lung recoil pressures at full inflation (required to measure FEVı) in females and at equivalent size, the airways of adult males would be wider than those of adult females (Martin et al., 1987).

Taking these data into account, FEVı, which is mainly dependent on bronchial caliber, would be negatively affected in females compared with the MVV, which is less affected in its expiratory phase by lung elastic recoil (Fairshter et al., 1989).

The MVV/FEVı ratio would then be higher in females, contrary to the results of our study and other data in the literature (Giske et al., 2003; Silva et al., 2020).

Therefore, further investigation is needed to explain this increase in the ratio in males.

In any case, determining the MVV/FEVı ratio without taking into account subjects sizes (Cid‐Juarez et al., 2017; Giske et al., 2003; Mohan‐Kumar & Gimenez, 1984), age (Cid‐Juarez et al., 2017), or racial origin (Cid‐Juarez et al., 2017; Giske et al., 2003; Mohan‐Kumar & Gimenez, 1984; Silva et al., 2020), could lead to an underestimation or overestimation of this value (Silva et al., 2020). Similarly, the use in clinical practice of a single MVV/FEVı ratio to calculate a subject's EVH TV (without taking gender into account) in the protocol recommended for athletes (Anderson et al., 2001; Koch et al., 2015; Spiering et al., 2004;) and in some guidelines (Levett et al., 2018; Takken et al., 2017), seems questionable because, according to our results, the influence of gender did not appear to be negligible.

Finally, our study, like that of many others who had, however, different reasons from ours (Fairshter et al., 1989; Harber et al., 1985), did not support the usual practice of estimating the MVV using a constant value of MVV/FEVı ratio, regardless the gender of the subject.

## 6 CONCLUSION

MVV/ FEVı ratio was statistically greater in males than in females.

Therefore, the use of the MVV estimated with a constant ratio without taking gender into account was, therefore, questionable. Since the cardiopulmonary function testing must be accurate, the healthcare professional should consider gender in any assessment that would use the estimated MVV in the CPET analysis (for ventilation limitation) or in EVH testing (to diagnose EIB).

Nevertheless, this study, whose results could have implications for spirometric and clinical practices, would benefit from being reinforced by other studies involving a larger population.

## CONFLICT OF INTEREST

The authors have no conflicts of interest.

## AUTHORS’ CONTRIBUTION

**Soualiho Ouattara**: Conception of the work, analysis, and interpretation of data for the work, drafting of the work, approved the final version of the manuscript, and agree to be accountable for all aspects of the work. **Edwige Siransy‐Balayssac**: Analysis, and interpretation of data for the work, drafting of the work, approved the final version of the manuscript, agree to be accountable for all aspects of the work. **Aya Liliane Kondo:** Acquisition of data for the work, drafting of the work, approved the final version of the manuscript, agree to be accountable for all aspects of the work. **Téniloh Augustin Yéo:** Acquisition of data for the work, drafting of the work, approved the final version of the manuscript, agree to be accountable for all aspects of the work. **Cyrille Serges Dah**: Revising the work critically for important intellectual content, approved the final version of the manuscript, agree to be accountable for all aspects of the work. **Pascal Bogui**: Revising the work critically for important intellectual content, approved the final version of the manuscript, agree to be accountable for all aspects of the work.
